# A novel paradigm for chronic subdural hematoma: regenerative medicine strategies targeting the pathological microenvironment

**DOI:** 10.3389/fcell.2026.1757623

**Published:** 2026-02-11

**Authors:** Zhijian Xu, Song Yang, Guifang Zheng, Huahui Chen, Xing Wan, Hu Xu, Yutiao Xu, Jian Wang, Minfeng Tong, Qi Tu

**Affiliations:** 1 Department of Neurosurgery, Jinhua Municipal Central Hospital, Jinhua, China; 2 Department of Neurosurgery, Jiaozhou Branch, East Hospital Affiliated to Tong ji University, Jiaozhou, China; 3 Department of Anesthesiology, Jinhua Municipal Central Hospital, Jinhua, China

**Keywords:** cSDH, microenvironment remodeling, pathological, regenerative medicine, targeted therapy

## Abstract

This review systematically summarizes the pathological microenvironment characteristics of chronic subdural hematoma (CSDH) and the regenerative medicine strategies for its intervention. CSDH is no longer regarded as a simple mechanical hematoma but is recognized as a dynamic pathological process driven by chronic inflammation, abnormal angiogenesis, extracellular matrix (ECM) imbalance, and interactions among immune cells. The article focuses on key cellular and molecular mechanisms within the microenvironment and highlights regulatory strategies targeting inflammation, vascular leakage, and matrix remodeling. These strategies include immunomodulation, stem cell therapy, exosome- and nanomaterial-based delivery systems. Such innovative approaches aim to restore tissue homeostasis at the biological level, advancing CSDH treatment from traditional surgical drainage toward microenvironment remodeling and functional reconstruction. They provide a theoretical basis for achieving precise and regenerative clinical therapies.

## 1 Introduction

**FIGURE 1:**
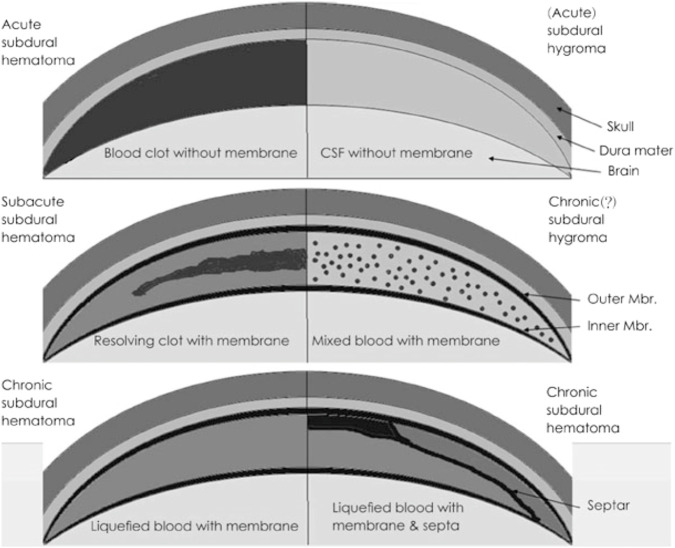
Pathological characteristics of subdural lesions. Modified from Lee KS. Natural history of chronic subdural haematoma. Brain Inj 18:351-358, 2004.31) Copyright 2004 by the Taylor and Francis. Reprinted with permission. CSF: cere brospinal fluid, Mbr.: membrane (Lee, 2015).

Chronic subdural hematoma (CSDH) is one of the most common chronic intracranial hemorrhagic disorders encountered in neurosurgical practice, and its incidence continues to rise, particularly in the context of accelerated global population aging (Chen et al., 2025a; Nouri et al., 2021; Lee, 2015; Weigel et al., 2022) (Figure 1). Although surgical interventions such as burr-hole drainage can rapidly relieve intracranial pressure and improve neurological function, a significant proportion of patients still experience postoperative recurrence, persistent hematoma expansion, or suboptimal long-term functional recovery (Rodriguez et al., 2023; Shibahashi, 2024). This suggests that simple mechanical evacuation does not fundamentally reverse the underlying biological processes driving disease chronicity. Recent studies have increasingly revealed that CSDH is not an inert “hematoma” in the traditional sense, but rather a complex pathological microenvironment characterized by chronic inflammation, persistent vascular leakage, aberrant fibrosis, and highly dynamic cell–matrix interactions (Rodriguez et al., 2023; Chen et al., 2025b; Nouri et al., 2021). The sustained activation of this microenvironment drives hematoma formation, maintenance, and recurrence (Huang et al., 2024).

Within this pathological microenvironment, fibroblasts, macrophages, and newly formed endothelial cells in the hematoma outer membrane interact and release a variety of inflammatory mediators, growth factors, and proteolytic enzymes, establishing a positive feedback loop of inflammation–vascular leakage–rebleeding (Edlmann et al., 2017). In addition, disruption of the extracellular matrix (ECM), imbalance in coagulation and fibrinolytic systems, and mechanical forces collectively contribute to disease progression (Huang et al., 2024). A deeper understanding of these mechanisms has redefined CSDH from a simple surgical disorder to a modifiable pathological condition driven by an abnormal microenvironment (Sahyouni et al., 2017).

Driven by rapid advances in regenerative medicine and biomaterials, strategies targeting the CSDH microenvironment have gradually become a research focus. These approaches include immunomodulation, stabilization of neovasculature, reconstruction of the extracellular matrix, promotion of repair using exosomes, and targeted delivery of drugs and signaling molecules via nanomaterials or biological scaffolds (Gao et al., 2019; Chen et al., 2022). Rather than merely aiming to “evacuate the hematoma,” these strategies focus on remodeling the microenvironment and restoring tissue homeostasis. They hold the potential to fundamentally reduce recurrence, enhance functional recovery, and advance CSDH treatment from conventional mechanical interventions toward biologically and regeneratively oriented therapies (Tamura et al., 2021; Sadeghian et al., 2023).

This review aims to systematically summarize the cellular and molecular characteristics of the CSDH microenvironment and to provide an overview of current innovative regenerative medicine therapies targeting microenvironmental regulation, refering to an integrated regenerative strategy encompassing immune modulation, vascular stabilization, and extracellular matrix reconstruction. It further explores their potential mechanisms, clinical applications, and translational challenges. Integrating these findings is expected to provide a theoretical foundation and practical guidance for developing more precise and effective comprehensive strategies for CSDH management.

## 2 Biological characteristics of the CSDH microenvironment

Recent multi-omics, histological, and *in vitro* studies have demonstrated that CSDH is sustained by a highly active pathological microenvironment (Edlmann et al., 2017; Zhong et al., 2024). This microenvironment is driven by specific cellular components, aberrant inflammatory networks, persistently leaky neovasculature, and a disordered extracellular matrix, collectively contributing to hematoma formation and recurrence (Kan et al., 2024). A detailed understanding of this microenvironment is critical for elucidating the disease progression, mechanisms of recurrence, and potential targets for regenerative medicine interventions in CSDH.

### 2.1 Cellular composition and dynamic interactions: the basis of the pathological ecosystem

#### 2.1.1 Outer membrane fibroblasts: structural and signaling hubs for hematoma maintenance

**FIGURE 2:**
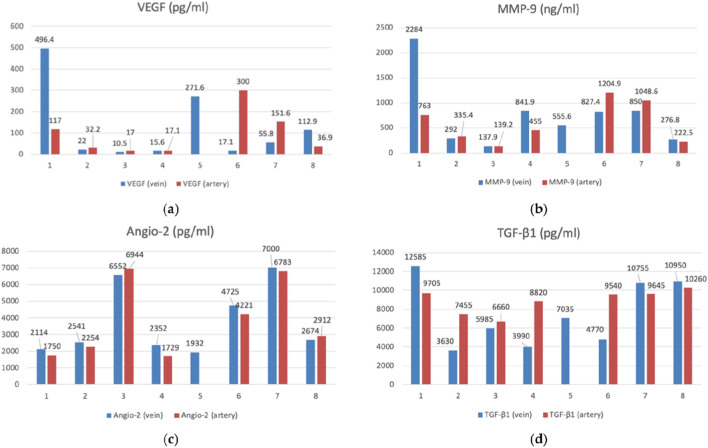
The bar graphs with datapoints show close trends in the concentrations of angiogenesis factors in the peripheral venous (n = 8) and arterial (n = 7) blood. **(a)** VEGF (vascular endothelial growth factor) (pg/mL), **(b)** MMP-9 (matrix metalloproteinase-9) (ng/mL), **(c)** Angio-2 (angiopoietin-2) (pg/mL), **(d)** TGF-β1 (transforming growth factor-β) (pg/mL) (Petrov et al., 2022).

The outer membrane of CSDH is rich in activated fibroblasts and myofibroblasts, which contribute not only to membrane thickening and extracellular matrix (ECM) remodeling but also to the secretion of numerous angiogenic factors and inflammatory mediators, such as VEGF, FGF-2, IL-6, and MMP-9 (Petrov et al., 2022; Hua et al., 2016; Cong et al., 2015) (Figure 2). These factors promote neovascularization, increase vascular permeability, and drive persistent fluid accumulation within the hematoma cavity. Single-cell transcriptomic studies indicate that CSDH fibroblasts exhibit a “regenerative-like activated” phenotype reminiscent of wound-healing tissue; however, their repair programs are aberrantly delayed and fail to terminate, resulting in a chronically active pathological membrane (Zhang et al., 2024).

#### 2.1.2 Macrophages and immune cell infiltration: central drivers of the inflammation–rebleeding loop

Macrophages are the predominant immune cells within the CSDH microenvironment, displaying a mixed M1/M2 phenotype (Yi et al., 2025). M1 macrophages amplify inflammation via TNF-α and IL-1β and induce endothelial injury, whereas M2 macrophages, despite their anti-inflammatory and reparative roles, can also promote angiogenesis and ECM degradation when excessively activated (Yi et al., 2025). Macrophage states in the CSDH microenvironment are highly plastic, existing along a spectrum rather than as discrete M1 or M2 phenotypes, with time-dependent transitions driven by local cytokine gradients, tissue injury signals, and microenvironmental remodeling. High concentrations of DAMPs in the hematoma fluid, such as HMGB1, can activate Toll-like receptors (TLRs), further enhancing macrophage immune activity and preventing proper resolution of inflammation (Osuka et al., 2020). Recent studies suggest that macrophage-derived exosomes carrying miRNAs, may contribute to the chronicity of the hematoma, representing potential targets for microenvironmental interventions.

#### 2.1.3 Endothelial cells: maintaining the abnormal leaky vascular network

**FIGURE 3:**
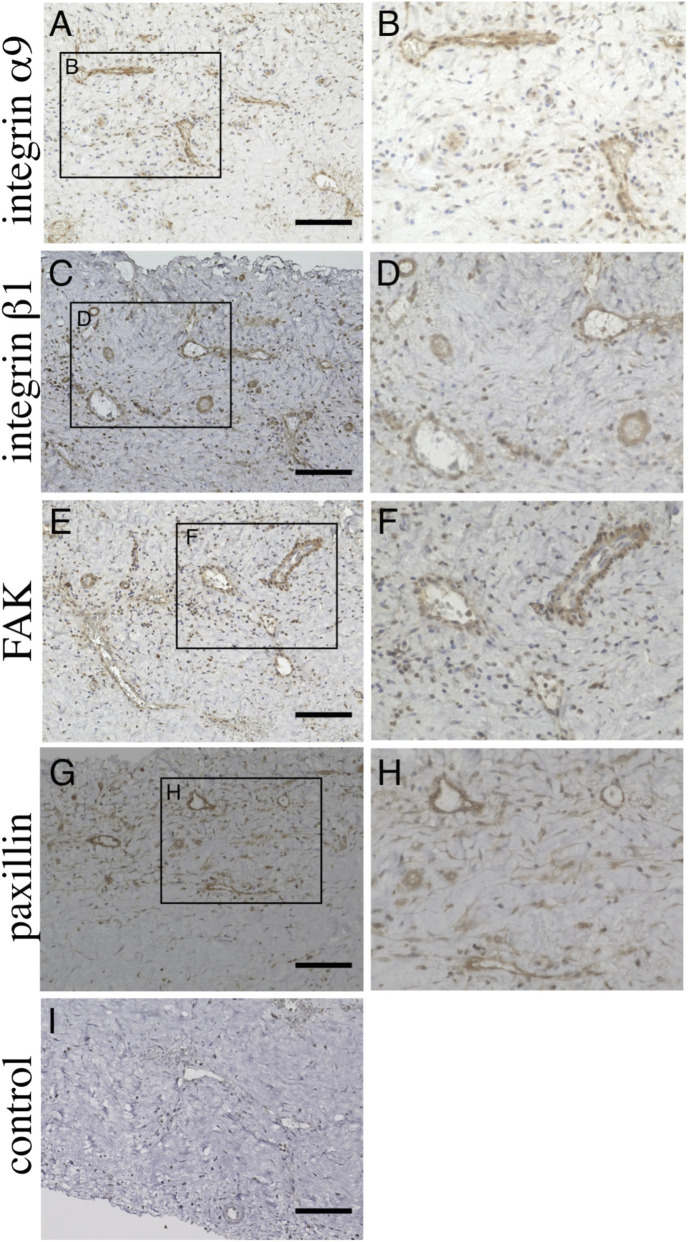
Immunohistochemical analysis of chronic subdural hematoma outer membrane. Ten-micrometer consecutive slices were immunostained with polyclonal antibodies against integ rin α9 **(A,B)**, integrin β1 **(C,D)**, focal adhesion kinase (FAK, **(E,F)** and paxillin **(G,H)** using the ABC method. The areas within the rectangle, labeled **(A,C,E,G)**, are shown at a higher magnification in **(A,B)**, integrin 1 **(C,D)**, focal adhesion kinase (FAK, **(E,F)** and paxillin **(G,H)** using the ABC method. The areas within the rectangle, labeled **(A,C,E,G)**, are shown at a higher magnification in Panels **(B,D,F,H)**, respectively. Note that these molecules are expressed in endothelial cells **(B,D,F,H)**. Slices immunostained without primary antibodies are shown in **(I)**. Scale bars = 100 µm (Osuka et al., 2023).

**FIGURE 4:**
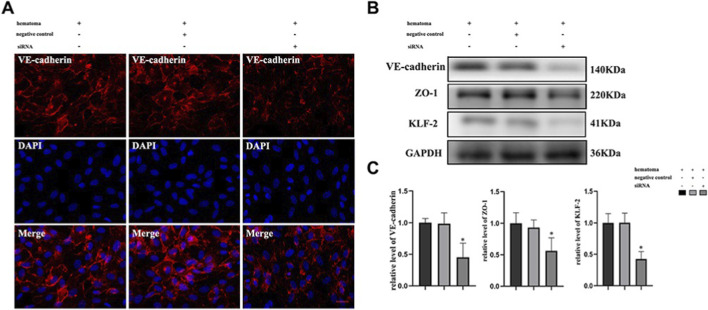
Hematoma sample stimulates cocultured cells and induces changes in the expression of tight junction proteins after knockdown of KLF-2 in endothelial cells. **(A)** The changes in tight junction proteins in endothelial cells after injury observed by immunofluorescence staining; **(B)** Western blot analysis of the changes in tight junction protein expression in endothelial cells with different genotypes after injury; **(C)**. Gray value analysis of panel **(B)** *P < 0.05 (Fan et al., 2020).

The CSDH outer membrane contains widespread, structurally incomplete, and functionally defective neovessels, which form a key pathological basis for hematoma chronicity (Yan et al., 2013; Osuka et al., 2023) (Figure 3). The vascular basement membrane in this region is generally fragmented or extremely thin, leading to significantly reduced mechanical stability of the vessel wall (Yamashima, 2000; Mehta et al., 2018). Concurrently, the expression of endothelial junction proteins, such as claudin-5 and occludin, is markedly downregulated, further compromising barrier integrity and increasing vascular permeability (Fan et al., 2020) (Figure 4). At the molecular level, overactivation of VEGF and Ang-2 generates strong pro-permeability angiogenic signals, causing these immature vessels to continuously leak exudates rich in inflammatory mediators and proteolytic enzymes, thereby sustaining hematoma expansion (Takei et al., 2021; Kim and Lee, 2023). Furthermore, dysfunction of the Tie2/Ang regulatory axis is considered a central mechanism underlying vascular instability. Recent animal studies have confirmed that reduced Tie2 signaling exacerbates endothelial barrier disruption and local rebleeding, playing a crucial role in maintaining the CSDH pathological state throughout its course (Isaji et al., 2020).

### 2.2 Molecular signaling networks: core regulatory axes driving chronic hematoma evolution

The formation and progression of CSDH are driven by multiple molecular signaling pathways, with inflammation amplification, abnormal angiogenesis, and coagulation–fibrinolysis imbalance forming the core regulatory axes supporting hematoma chronicity.

First, regarding inflammatory signaling, NF-κB and its upstream TLR4 pathway are persistently activated in the outer membrane, resulting in high-level expression of pro-inflammatory cytokines such as IL-6, IL-8, and TNF-α, thereby establishing a typical chronic inflammation amplification loop (Osuka et al., 2020; Holl et al., 2018). Simultaneously, concurrent activation of the IL-6/STAT3 pathway not only promotes continuous thickening of the outer membrane but also closely interacts with abnormal angiogenesis, representing a key molecular basis for the high recurrence propensity of CSDH (Osuka et al., 2017; Osuka et al., 2013). Evidence supporting activation of this inflammatory–angiogenic positive feedback loop is derived primarily from immunohistochemical analyses of CSDH outer membranes, which demonstrate increased NF-κB nuclear translocation and STAT3 phosphorylation, as well as from experimental animal models showing attenuation of membrane growth following pathway inhibition.

**FIGURE 5:**
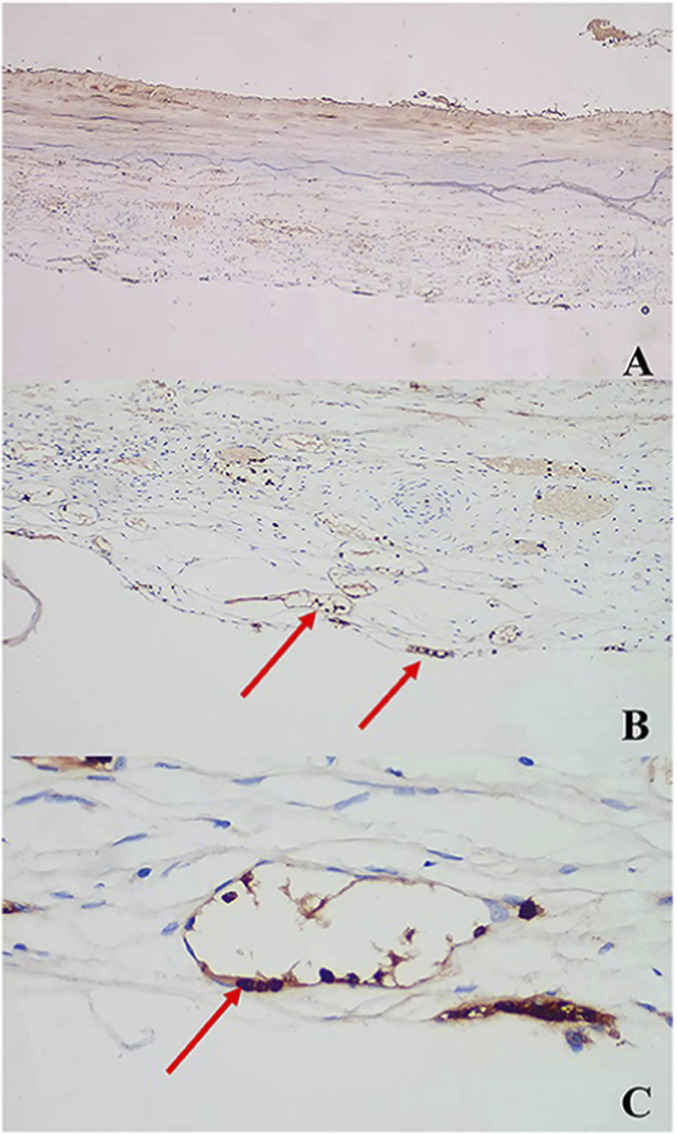
AntiMMP antibody immunostaining: **(A)** ×, **(B)** ×, **(C)** × (arrows) (Su et al., 2022b).

In terms of angiogenic regulation, dual upregulation of VEGF and Ang-2 constitutes the molecular basis for the “leaky angiogenesis” phenotype observed in CSDH (Kim and Lee, 2023). Elevated VEGF drives the formation of numerous structurally immature neovessels, while Ang-2 antagonizes Tie2-mediated stabilization signals, further compromising vascular integrity and increasing susceptibility to rupture and leakage. Additionally, overexpression of matrix metalloproteinases such as MMP-9 accelerates degradation of the vascular basement membrane, maintaining long-term vascular instability and serving as a critical source of repeated microbleeds and hematoma expansion (Su et al., 2022a; Su et al., 2022b) (Figure 5).

At the coagulation–fibrinolysis level, the hematoma cavity exhibits a characteristic “hyperfibrinolytic and hypocoagulable” state. Tissue plasminogen activator (tPA) is significantly elevated, while abnormal fluctuations in PAI-1 further exacerbate uncontrolled fibrinolytic activity (Brazdzioni et al., 2019; Neils et al., 2012; Doan et al., 2018). Repeated local microbleeds generate degradation products under persistent fibrinolysis, which in turn enhance inflammatory responses and promote extracellular matrix destruction, forming a bidirectional pathological cycle between inflammation and fibrinolysis, and creating the unique “hemorrhagic microenvironment” of CSDH.

Overall, inflammation amplification, abnormal angiogenesis, and coagulation–fibrinolysis dysregulation collectively establish a self-sustaining and difficult-to-terminate pathological signaling network, representing the key mechanisms driving the persistence and recurrence of CSDH.

### 2.3 Extracellular matrix (ECM) and biophysical factors: key microenvironmental foundations regulating cellular behavior

The extracellular matrix (ECM) and biophysical factors play critical regulatory roles in the CSDH microenvironment, influencing not only cellular behaviors but also the tissue’s ability to restore homeostasis from the ongoing inflammatory and hemorrhagic cycles (Huang et al., 2024; Huang et al., 2024). The ECM of the CSDH outer membrane exhibits marked structural remodeling, characterized by disorganized collagen fibers, abundant deposition of fibronectin and fibrin degradation products, and overexpression of MMP-2 and MMP-9, leading to continuous ECM degradation and reconstruction (Hua et al., 2015). This abnormal dynamic imbalance places local tissue in a state resembling a “chronic wound,” affecting fibroblast proliferation, migration, and secretory activity, while also promoting immune cell infiltration and compromising vascular stability, thus forming a key structural basis for sustaining the pathological CSDH cycle.

Beyond the chemical and structural properties of the ECM, various biophysical factors within the hematoma cavity also profoundly influence microenvironmental evolution. As the hematoma enlarges, increased intracavitary pressure directly compresses neovessels in the outer membrane, further enhancing vascular permeability and promoting the influx of exudates and inflammatory mediators, thereby creating a vicious cycle (Mehta et al., 2018). Concurrently, mechanical stretching of local tissue can induce fibroblasts to adopt a more secretory phenotype, releasing large amounts of pro-angiogenic factors and further driving the formation of unstable neovessels (Mehmandoost et al., 2025). Recent studies also suggest that changes in shear stress and osmotic pressure may affect endothelial junctional structures and barrier function, providing a biological basis for repeated leakage and recurrence in CSDH.

Overall, abnormal ECM remodeling and biophysical mechanical stimuli together constitute key factors regulating cellular behavior in the CSDH microenvironment. They maintain local tissue in a persistent state of inflammation, leakage, and failed repair, representing important targets for future microenvironmental remodeling and regenerative therapies.

## 3 Regenerative medicine interventions targeting the CSDH microenvironment

### 3.1 Anti-inflammatory and immune remodeling strategies

The pathological progression of CSDH is closely linked to persistent inflammatory activation and abnormal immune cell polarization. Long-term activation of signaling pathways such as NF-κB and IL-6/STAT3 in the membrane tissue increases pro-inflammatory cytokine release, enhances angiogenesis, and drives continuous membrane thickening (Osuka et al., 2017; Osuka et al., 2016). Therefore, inhibiting these key pathways has emerged as an important strategy to regulate the local microenvironment. Corticosteroids, atorvastatin, and other drugs have been shown to downregulate these pathways, attenuating the inflammatory cascade in membrane tissue and reducing the risk of hematoma recurrence. Recently, precise regulation of the immune microenvironment has become a research focus. Strategies that promote macrophage polarization from the pro-inflammatory M1 phenotype to the reparative M2 phenotype show significant potential. Various miRNAs, including miR-21 and miR-146a, can regulate macrophage polarization and suppress pro-inflammatory signaling, reducing local inflammation and promoting tissue repair in animal models, providing a feasible direction for future immune-targeted therapies (Wang et al., 2018; Olivieri et al., 2021; Böttcher et al., 2023).

### 3.2 Inhibition of abnormal angiogenesis and vascular leakage

The persistent growth and recurrence of CSDH are closely associated with fragile and abnormal neovasculature. Pathological vessels often exhibit high VEGF expression, basement membrane defects, and reduced endothelial junction integrity, leading to increased vascular permeability and accumulation of fluid within the hematoma cavity. Anti-VEGF or multi-target anti-angiogenic therapies have been shown in animal models to reduce fragile vessel density, inhibit leakage, and attenuate inflammatory cascades (Verma et al., 2024). Additionally, restoring the Tie2/Ang signaling axis enhances endothelial barrier integrity, reducing the formation of leaky vessels (Isaji et al., 2020). Small molecules or protease inhibitors targeting basement membrane degradation can further stabilize vascular structures and interrupt the “inflammation–leakage–re-inflammation” cycle. Integrated strategies that modulate angiogenesis and vascular barrier function hold promise for fundamentally reducing CSDH recurrence rates.

### 3.3 Extracellular vesicle (EV) and RNA delivery-based therapies

Advances in nanomedicine have opened new avenues for extracellular vesicles (EV) - and nucleic acid-based strategies to modulate the CSDH microenvironment. EVs can carry miRNAs, siRNAs, and proteins, playing important roles in intercellular communication and inflammation regulation. By delivering specific miRNAs or siRNAs to target pro-inflammatory pathways, inhibit vascular leakage, or modulate matrix-degrading factors, multidimensional molecular-level interventions can be achieved in membrane pathology (Gao et al., 2019). Concurrently, nanocarrier technologies have greatly enhanced the stability and targeting of these biomolecules. DNA nanostructures such as tetrahedral framework nucleic acids (tFNA) exhibit high biocompatibility, efficient cellular uptake, and the ability to cross the blood-brain barrier, and have demonstrated anti-inflammatory and repair-promoting effects in neural disorders (Fu et al., 2021). Applying tFNA platforms to deliver anti-inflammatory, anti-leakage, or repair-related RNAs could enable precise and synergistic regulation of the CSDH microenvironment, providing a foundation for highly controllable regenerative medicine therapies in the future.

Despite the promising potential of regenerative medicine–based strategies for modulating the chronic subdural hematoma microenvironment, several limitations and risks should be acknowledged. Therapeutic responses may be heterogeneous among patients due to differences in age, comorbidities, hematoma stage, and underlying microenvironmental profiles. In addition, the efficiency and precision of local or targeted delivery—particularly for extracellular vesicles, nucleic acid–based therapeutics, and biomaterials—remain technically challenging, potentially limiting their reproducibility and translational applicability. Safety considerations, including immunogenicity, off-target effects, long-term biocompatibility, and dose control, also require careful evaluation before widespread clinical adoption. Addressing these challenges through optimized delivery systems, rigorous preclinical validation, and well-designed clinical studies will be essential for translating regenerative microenvironmental regulation from concept to clinical reality.

## 4 Integrated microenvironment remodeling model: from lesion clearance to functional reconstruction

With the deepening understanding of CSDH pathophysiology and dynamic microenvironmental evolution, traditional surgical drainage alone no longer meets the clinical demands for low recurrence rates and functional restoration (Edlmann et al., 2017; Shlobin et al., 2021). Emerging studies have gradually combined “lesion clearance” with “microenvironmental regenerative repair” to establish a multi-layered, integrated treatment model aimed at restoring homeostasis and reconstructing tissue function.

### 4.1 Combined “surgery + microenvironment regulation” approach

Surgical drainage can rapidly reduce hematoma volume, relieve local pressure, and provide a foundation for rebalancing the microenvironment (Uno, 2023). However, residual inflammatory cells, abnormal vessels, and actively degrading extracellular matrix (ECM) in the outer membrane may still drive postoperative recurrence (Hamou et al., 2022). Accordingly, middle meningeal artery (MMA) embolization has emerged as a therapeutic approach. By occluding the abnormal neovasculature that supplies the pathological membranes of the subdural space, it reduces vascular leakage and inflammatory drive, thereby effectively lowering the recurrence rate of chronic subdural hematoma (Schmolling et al., 2024; Catapano et al., 2020). On the other hand, perioperative interventions using targeted drugs or regenerative materials—such as anti-inflammatory molecules, biologics that inhibit vascular leakage, or hydrogels capable of sustained release of reparative factors—can further suppress inflammatory cascades, improve vascular stability, and promote orderly remodeling of membrane tissue, potentially lowering recurrence rates. Experimental studies have shown that delivering anti-inflammatory or anti-fibrinolytic agents directly into the drainage cavity or applying them to the outer membrane surface can significantly enhance tissue repair, supporting the concept of continuous intra- and postoperative microenvironmental regulation.

### 4.2 “Non-surgical” regenerative therapies

With advances in regenerative medicine and nanomaterials, non-surgical regenerative therapies are gradually emerging. This approach aims to precisely modulate core pathological elements of the microenvironment, promoting spontaneous hematoma absorption and preventing expansion without surgical intervention (Yun and Ding, 2020; Scerrati et al., 2020). Its theoretical basis includes: reducing exudate formation by inhibiting central inflammatory pathways, decreasing rebleeding by stabilizing fragile neovessels, and restoring tissue homeostasis by reconstructing ECM architecture and balancing protease activity. Injectable hydrogels, targeted RNA nanomedicines, MSC-derived extracellular vesicles, and multifunctional biomaterials may all serve as non-invasive or minimally invasive delivery modalities, offering particular value for elderly patients or those with comorbidities who cannot tolerate surgery (Varderidou-Minasian and Lorenowicz, 2020). Preclinical models have shown that combined interventions targeting inflammation, vascular stabilization, and ECM reconstruction can significantly reduce hematoma volume without surgical drainage, providing a scientific basis for non-surgical regenerative therapy.

### 4.3 Precision regeneration

Advances in precision regenerative medicine offer a higher-level theoretical framework for integrated CSDH treatment. Multi-dimensional analyses—including hematoma fluid omics, outer membrane transcriptomics, proteomics, and single-cell sequencing—can reveal patient-specific heterogeneity in inflammation, angiogenesis, ECM degradation, and immune responses (Zhang et al., 2024). This heterogeneity suggests that CSDH is not a single disease entity but may comprise multiple microenvironment-driven subtypes, such as inflammation-dominant, vascular leakage-dominant, or fibrosis/ECM imbalance-dominant types (Edlmann et al., 2017). Subtype-based therapeutic models enable more precise interventions: inflammation-dominant patients may benefit from NF-κB or IL-6/STAT3 inhibitors; vascular leakage-dominant patients may be better suited for Tie2 activators, anti-VEGF therapy, or protease inhibitors; ECM imbalance-dominant patients may benefit more from MSCs or biomaterials with matrix-reconstructive capabilities (Osuka et al., 2020; Osuka et al., 2017; Jensen et al., 2024). In the future, integrating clinical imaging, omics data, and AI-based predictive models may facilitate individualized treatment pathways, achieving true precision management of the CSDH microenvironment—from lesion clearance to functional reconstruction.

## 5 Challenges and future perspectives

Despite significant advances in understanding the CSDH microenvironment and developing regenerative medicine interventions, translating these findings from basic research to clinical application remains challenging. Careful consideration of future directions is essential, given the incomplete theoretical framework and the need to optimize technical approaches.

First, microenvironmental research itself presents substantial technical hurdles, particularly in a dynamically evolving disease such as CSDH. Sampling of hematoma cavities and outer membrane tissue typically relies on surgical access, which limits sample size and makes it difficult to capture continuous dynamic changes across different disease stages. Existing animal and *in vitro* models also have significant limitations: most animal models cannot fully recapitulate the chronic evolution of human CSDH, especially in terms of brain anatomy, immune responses, and angiogenic patterns; *in vitro* models lack the complex mechanical forces and multicellular interactions present in the true microenvironment. Therefore, there is an urgent need to develop clinically relevant 3D organoid models, bioprinted membrane structures, or controllable microfluidic chip systems to more accurately reconstruct the CSDH pathological microenvironment (Kellogg et al., 2021; Chen et al., 2024; Kono, 2025).

Second, challenges remain regarding the safety and efficacy of biomaterials and stem cell therapies. Biomaterial degradation rates, biocompatibility of degradation products, potential toxicity, and inflammation or immune activation induced by material implantation may affect therapeutic outcomes or lead to adverse events. Stem cell therapy faces additional constraints, including source heterogeneity, purification difficulties, batch variability, and ethical and regulatory issues; long-term safety concerns, such as immune rejection, abnormal differentiation, or tumorigenicity, require extended follow-up data (Loyo et al., 2007; Yokobori et al., 2019; Kannan et al., 2002). Extracellular vesicle (EV) and RNA-based therapies, while offering precise regulation, still face technical challenges in dose control, *in vivo* stability, immune barrier penetration, targeting efficiency, and scalable manufacturing. Enhancing delivery system targeting while minimizing off-target distribution is key for future clinical translation.

Third, clinical translation and regulatory requirements for CSDH regenerative therapies pose new challenges. Designing clinical trials necessitates careful consideration of therapeutic windows, delivery methods, dosing regimens, and safety assessments. Since CSDH patients are often elderly with multiple comorbidities, trial design becomes further complicated. Identifying patient populations suitable for regenerative therapy, establishing standardized inclusion/exclusion criteria, and optimizing combined surgical and regenerative approaches all require substantial clinical evidence. Moreover, the high cost of regenerative medicine products may limit accessibility and economic feasibility, representing an additional barrier to clinical adoption.

Finally, interdisciplinary integration will be a critical driver of innovation in microenvironment regulation and regenerative therapy for CSDH. Collaboration among neurosurgery, regenerative medicine, nanomaterials, immunology, tissue engineering, and bioinformatics can provide comprehensive support for deciphering complex pathological mechanisms and developing novel therapeutic strategies. For example, nanomaterials can enhance the targeting of RNA and EV-based therapies; regenerative medicine can provide structural support for hematoma membrane repair; and immunology can guide precise regulation of the immune microenvironment. Establishing multi-dimensional bioinformatic databases integrating imaging, omics, clinical outcomes, and pathological features will further enable molecular subtyping of the CSDH microenvironment, laying the foundation for precision interventions and individualized treatment strategies.

In summary, although regenerative medicine for CSDH faces numerous challenges, technological advancements and interdisciplinary collaboration hold immense potential. These developments may shift treatment paradigms from simple lesion removal to comprehensive microenvironment regulation and functional tissue reconstruction, achieving truly precise and regenerative therapy.

## 6 Conclusion

CSDH is no longer regarded as a purely mechanical hematoma, but rather as a chronic disease driven by a pathological microenvironment, whose formation, maintenance, and recurrence rely on the long-term, coordinated action of multiple factors, including inflammation amplification, abnormal angiogenesis, ECM imbalance, and dynamic interactions among immune cells. This paradigm shift provides a theoretical basis for innovative therapeutic strategies, suggesting that microenvironmental remodeling interventions can fundamentally alter hematoma progression and reduce recurrence risk. Current evidence shows that strategies targeting the CSDH microenvironment from multiple angles can work together to improve local pathology. These approaches include immune regulation, vascular stabilization, ECM reconstruction, regenerative therapies using stem cells or extracellular vesicles, and biomaterial-assisted interventions. Together, they provide new opportunities to restore outer membrane integrity, stabilize blood vessels, and recover tissue function.

The application of regenerative medicine and microenvironmental engineering is driving a shift in CSDH treatment from conventional “mechanical drainage” toward an integrated approach that combines biological and regenerative interventions. This transition emphasizes not only intraoperative hematoma clearance and pressure relief, but also precise regulation of the microenvironment to achieve membrane remodeling, inflammation suppression, and functional recovery, laying the foundation for future individualized and precision therapy. Nevertheless, translating these innovative strategies into clinical practice requires further high-quality studies to validate their safety, efficacy, and feasibility, alongside the development of standardized treatment protocols and evaluation frameworks. Through the continuous integration of basic research and clinical trials, microenvironmental remodeling and regenerative medicine hold the potential to establish a new paradigm for CSDH treatment, achieving comprehensive management from lesion removal to functional reconstruction.

## References

[B1] Böttcher S. Kalies K. Knöpp K. Dutzmann J. Müller S. Hüttelmaier S. (2023). Circular decoys sponge miR-21 and miR-146a in senescent vascular cells. Atherosclerosis 379, S30–S31. 10.1016/j.atherosclerosis.2023.06.147

[B2] Brazdzionis J. Patchana T. Wiginton J. G. t. Wacker M. R. Menoni R. Miulli D. E. (2019). Intracatheter tissue plasminogen activator for chronic subdural hematomas after failed bedside twist drill craniostomy: a retrospective review. Cureus 11 (12), e6472. 10.7759/cureus.6472 32025399 PMC6984181

[B3] Catapano J. S. Nguyen C. L. Wakim A. A. Albuquerque F. C. Ducruet A. F. (2020). Middle meningeal artery embolization for chronic subdural Hematoma. Front. Neurology 11, 557233. 10.3389/fneur.2020.557233 33192990 PMC7606850

[B4] Chen R. Wen D. Fu W. Xing L. Ma L. Liu Y. (2022). Treatment effect of DNA framework nucleic acids on diffuse microvascular endothelial cell injury after subarachnoid hemorrhage. Cell Proliferation 55 (4), e13206. 10.1111/cpr.13206 35187748 PMC9055902

[B5] Chen H. Colasurdo M. Malhotra A. Gandhi D. Bodanapally U. K. (2024). Advances in chronic subdural hematoma and membrane imaging. Front. Neurology 15, 1366238. 10.3389/fneur.2024.1366238 38725642 PMC11079242

[B6] Chen H. Colasurdo M. Bodanapally U. K. Malhotra A. Gandhi D. (2025a). Chronic subdural hematoma: a review of Current knowledge, treatment modalities, and clinical trials of middle meningeal artery embolization, Chronic Subdural Hematoma, 15(4) e200501. 10.1212/CPJ.0000000000200501 40678273 PMC12264714

[B7] Chen H. Colasurdo M. Bodanapally U. K. Malhotra A. Gandhi D. (2025b). Chronic subdural hematoma: a review of Current knowledge, treatment modalities, and clinical trials of middle meningeal artery embolization, neurology. Clin. Practice 15 (4), e200501. 10.1212/cpj.0000000000200501 40678273 PMC12264714

[B8] Cong H. Gang Z. Yan F. Hongyan Y. Hongmei S. Li B. J. J. N. (2015). Role of matrix Metalloproteinase-2, matrix Metalloproteinase-9, and vascular endothelial growth factor in the development of chronic. Subdural Hematoma 33 (1). 10.1089/neu.214.3724 PMC4700393 25646653

[B9] Doan N. B. Patel M. Nguyen H. S. Doan H. Montoure A. Shabani S. (2018). Safe use of tissue plasminogen activator in conjunction with the Integra camino bolt for the drainage of chronic subdural Hematoma. Asian Journal Neurosurgery 13 (2), 485–486. 10.4103/ajns.AJNS_230_16 29682068 PMC5898139

[B10] Edlmann E. Giorgi-Coll S. Whitfield P. C. Carpenter K. L. H. Hutchinson P. J. (2017). Pathophysiology of chronic subdural haematoma: inflammation, angiogenesis and implications for pharmacotherapy. J. Neuroinflammation 14 (1), 108. 10.1186/s12974-017-0881-y 28558815 PMC5450087

[B11] Fan Y. Wang D. Rao C. Li Y. Rong H. Wang Z. (2020). Atorvastatin combined with low-dose dexamethasone treatment protects endothelial function impaired by chronic subdural hematoma *via* the transcription factor KLF-2, drug design. Development Therapy 14, 3291–3299. 10.2147/dddt.S256050 32848367 PMC7429211

[B12] Fu W. Ma L. Ju Y. Xu J. Li H. Shi S. (2021). Therapeutic siCCR2 loaded by tetrahedral framework DNA nanorobotics in therapy for intracranial hemorrhage. Adv. Funct. Mater. 31 (33), 2170239. 10.1002/adfm.202170239

[B13] Gao C. Gong Z. Wang D. Huang J. Qian Y. Nie M. (2019). Hematoma-derived exosomes of chronic subdural hematoma promote abnormal angiogenesis and inhibit hematoma absorption through miR-144-5p. Aging 11 (24), 12147–12164. 10.18632/aging.102550 31841443 PMC6949077

[B14] Hamou H. A. Clusmann H. Schulz J. B. Wiesmann M. Altiok E. Höllig A. (2022). Chronic subdural hematoma. Dtsch. Arzteblatt International 119 (12), 208–213. 10.3238/arztebl.m2022.0144 35236548 PMC9277133

[B15] Holl D. C. Volovici V. Dirven C. M. F. Peul W. C. van Kooten F. Jellema K. (2018). Pathophysiology and nonsurgical treatment of chronic subdural hematoma: from past to present to future, World Neurosurgery 116 402–411. 10.1016/j.wneu.2018.05.037 29772364

[B16] Hua C. Feng Y. Yuan Y. Song M. Bie L. (2015). Role of MMP-2, MMP-9 and VEGF in the development of chronic subdural hematoma. J. Neurotrauma 33. 10.1089/neu.2014.3724 PMC4700393 25646653

[B17] Hua C. Zhao G. Feng Y. Yuan H. Song H. Bie L. (2016). Role of matrix Metalloproteinase-2, matrix Metalloproteinase-9, and vascular endothelial growth factor in the development of chronic subdural Hematoma. J. Neurotrauma 33 (1), 65–70. 10.1089/ne.2014.3724 25646653 PMC4700393

[B18] Huang M. Dai J. Zhong X. Wang J. Xu J. DU B. (2024). The pathogenesis of chronic subdural hematoma in the perspective of neomembrane formation and related mechanisms. Biocell 48 (6), 889–896. 10.32604/biocell.2024.050097

[B19] Isaji T. Osuka K. Ohmichi Y. Ohmichi M. Naito M. Nakano T. (2020). Expression of angiopoietins and angiogenic signaling pathway molecules in chronic subdural hematomas. J. Neurotrauma 37 (23), 2493–2498. 10.1089/neu.2020.7042 32458767

[B20] Jensen T. S. R. Thiesson E. M. Fugleholm K. Wohlfahrt J. Munch T. N. (2024). Inflammatory risk factors for chronic subdural hematoma in a nationwide cohort. J. Inflammation Research 17, 8261–8270. 10.2147/jir.S472849 39525314 PMC11550703

[B21] Kan P. Fiorella D. Dabus G. Samaniego E. A. Lanzino G. Siddiqui A. H. (2024). ARISE I consensus statement on the management of chronic subdural Hematoma. Stroke 55 (5), 1438–1448. 10.1161/strokeaha.123.044129 38648281

[B22] Kannan K. Koh L. P. Linn Y. C. (2002). Subdural hematoma in two hematopoietic stem cell transplant patients with post-dural puncture headache and initially normal CT brain scan. Ann. Hematology 81 (9), 540–542. 10.1007/s00277-002-0496-x 12373359

[B23] Kellogg R. T. Vargas J. Barros G. Sen R. Bass D. Mason J. R. (2021). Segmentation of chronic subdural hematomas using 3D convolutional neural networks. World Neurosurgery 148, e58–e65. 10.1016/j.wneu.2020.12.014 33359736

[B24] Kim K. H. Lee Y. (2023). Medical management of chronic subdural Hematoma. Korean Journal Neurotrauma 19 (3), 288–297. 10.13004/kjnt.2023.19.e47 37840605 PMC10567532

[B25] Kono K. (2025). E-153 real-time AI-assisted MMA embolization for subdural hematoma: a preliminary experience. J J. NeuroInterventional Surg. 17 (Suppl. 1), A195–A196. 10.1136/jnis-2025-SNIS.268

[B26] Lee K. S. (2015). History of chronic subdural Hematoma. Korean Journal Neurotrauma 11 (2), 27–34. 10.13004/kjnt.2015.11.2.27 27169062 PMC4847516

[B27] Loyo M. Quintanilla-Dieck Mde L. Stein B. Bolaños-Meade J. (2007). Bilateral subdural hematoma after hematopoietic stem cell transplantation: a diagnosis often overlooked. Leukemia and Lymphoma 48 (4), 835–836. 10.1080/10428190601175385 17454650

[B28] Mehmandoost M. Bahri A. Hasheminejad A. Sharifi H. Oveisi S. Fahim F. (2025). Prevalence, etiology, risk factors, management options, and outcomes in chronic subdural hematoma (cSDH): a comprehensive literature review of recent advances, Brain Disord. 19 100271. 10.1016/j.dscb.2025.100271

[B29] Mehta V. Harward S. C. Sankey E. W. Nayar G. Codd P. J. (2018). Evidence based diagnosis and management of chronic subdural hematoma: a review of the literature, J. Clin. Neurosci. 50 7–15. 10.1016/j.jocn.2018.01.050 29428263

[B30] Neils D. M. Singanallur P. S. Wang H. Tracy P. Klopfenstein J. Dinh D. (2012). Recurrence-Free chronic subdural hematomas: a retrospective analysis of the instillation of tissue plasminogen activator in addition to twist drill or burr hole drainage in the treatment of chronic subdural hematomas, World Neurosurgery 78(1) 145–149. 10.1016/j.wneu.2011.08.032 22120294

[B31] Nouri A. Gondar R. Schaller K. Meling T. (2021). Chronic Subdural Hematoma (cSDH): a review of the current state of the art. Brain and Spine 1, 100300. 10.1016/j.bas.2021.100300 36247395 PMC9560707

[B32] Olivieri F. Prattichizzo F. Giuliani A. Matacchione G. Rippo M. R. Sabbatinelli J. (2021). miR-21 and miR-146a: the microRNAs of inflammaging and age-related diseases, Ageing Res. Rev. 70 101374. 10.1016/j.arr.2021.101374 34082077

[B33] Osuka K. Watanabe Y. Usuda N. Atsuzawa K. Shima H. Takeuchi M. (2013). Activation of JAK-STAT3 signaling pathway in chronic subdural hematoma outer membranes. Neurosci. Letters 534, 166–170. 10.1016/j.neulet.2012.11.011 23174178

[B34] Osuka K. Watanabe Y. Usuda N. Aoyama M. Kawaguchi R. Watabe T. (2016). Activation of signal transducer and activator of transcription 3 in endothelial cells of chronic subdural Hematoma outer membranes, World Neurosurgery 91 376–382. 10.1016/j.wneu.2016.04.025 27102635

[B35] Osuka K. Watanabe Y. Usuda N. Aoyama M. Iwami K. Takeuchi M. (2017). Inhibitory mechanism of the outer membrane growth of chronic subdural hematomas. J. Neurotrauma 34 (11), 1996–2000. 10.1089/neu.2016.4623 28027695

[B36] Osuka K. Watanabe Y. Usuda N. Iwami K. Miyachi S. Takayasu M. (2020). Expression of high mobility group B1 and toll-like receptor-nuclear factor κB signaling pathway in chronic subdural hematomas. PloS One 15 (6), e0233643. 10.1371/journal.pone.0233643 32479555 PMC7263617

[B37] Osuka K. Ohmichi Y. Ohmichi M. Honma S. Suzuki C. Aoyama M. (2023). Angiogenesis in the outer membrane of chronic subdural hematomas through thrombin-cleaved osteopontin and the integrin α9 and integrin β1 signaling pathways. Biomedicines 11 (5), 1440. 10.3390/biomedicines11051440 37239111 PMC10216439

[B38] Petrov A. Ivanov A. Dryagina N. Petrova A. Samochernykh K. Rozhchenko L. (2022). Angiogenetic factors in chronic subdural Hematoma Development. Diagn. Basel, Switz. 12 (11), 2787. 10.3390/diagnostics12112787 36428849 PMC9689028

[B39] Rodriguez B. Morgan I. Young T. Vlastos J. Williams T. Hrabarchuk E. I. (2023). Surgical techniques for evacuation of chronic subdural hematoma: a mini-review. Front. Neurology 14, 1086645. 10.3389/fneur.2023.1086645 37456631 PMC10338715

[B40] Sadeghian H. Chida K. Motiei-Langroudi R. (2023). Editorial: chronic subdural hematoma: overview of recent therapeutic advancements. Front. Neurology 14, 1155680. 10.3389/fneur.2023.1155680 37077574 PMC10106743

[B41] Sahyouni R. Goshtasbi K. Mahmoodi A. Tran D. K. Chen J. W. (2017). Chronic subdural hematoma: a historical and clinical perspective. World Neurosurgery 108, 948–953. 10.1016/j.wneu.2017.09.064 28935548 PMC12747318

[B42] Scerrati A. Visani J. Ricciardi L. Dones F. Rustemi O. Cavallo M. A. (2020). To drill or not to drill, that is the question: nonsurgical treatment of chronic subdural hematoma in the elderly. A systematic review. Neurosurg. Focus 49 (4), E7. 10.3171/2020.7.Focus20237 33002869

[B43] Schmolling Á. H. Pérez-García C. Trejo C. López-Frías A. Jaroenngarmsamer T. Rosati S. (2024). Middle meningeal artery embolization for management of chronic. Subdural Hematoma 44 (4), e230158. 10.1148/rg.230158 38451847

[B44] Shibahashi K. (2024). Optimising treatment for chronic subdural haematoma. Lancet 403 (10446), 2757–2759. 10.1016/S0140-6736(24)00879-1 38852599

[B45] Shlobin N. A. Kedda J. Wishart D. Garcia R. M. Rosseau G. (2021). Surgical management of chronic subdural hematoma in older adults: a systematic review. Journals Gerontology. Ser. A, Biol. Sciences Medical Sciences 76 (8), 1454–1462. 10.1093/gerona/glaa293 33220683 PMC8277090

[B46] Su G. J. Gao J. Wu C. W. Zou J. F. Zhang D. Zhu D. L. (2022a). Serum levels of MMP-8 and MMP-9 as markers in chronic subdural Hematoma. J. Clinical Medicine 11 (4), 902. 10.3390/jcm11040902 35207175 PMC8878690

[B47] Su G. J. Zhang D. Wu J. N. Deng Y. H. Wu C. W. Zhang X. J. (2022b). Immunoexpression of MMP-8 and MMP-9 in chronic subdural hematoma. Front. Neurology 13, 988854. 10.3389/fneur.2022.988854 36061997 PMC9428760

[B48] Takei J. Tanaka T. Yamamoto Y. Hatano K. Ichinose D. Maruyama F. (2021). Significantly high concentrations of vascular endothelial growth factor in chronic subdural hematoma with trabecular formation, Clin. Neurology Neurosurg. 202 106458. 10.1016/j.clineuro.2020.106458 33545457

[B49] Tamura R. Sato M. Yoshida K. Toda M. (2021). History and current progress of chronic subdural hematoma. J. Neurological Sciences 429, 118066. 10.1016/j.jns.2021.118066 34488045

[B50] Uno M. (2023). Chronic subdural hematoma-evolution of etiology and surgical treatment. Neurol. Medico-Chirurgica 63 (1), 1–8. 10.2176/jns-nmc.2022-0207 36288974 PMC9894619

[B51] Varderidou-Minasian S. Lorenowicz M. J. (2020). Mesenchymal stromal/stem cell-derived extracellular vesicles in tissue repair: challenges and opportunities. Theranostics 10 (13), 5979–5997. 10.7150/thno.40122 32483432 PMC7254996

[B52] Verma Y. Abdelghaffar M. Verma O. Gajjar A. Ghozy S. Kallmes D. F. (2024). “Bevacizumab: the future of chronic subdural hematoma,” in Interventional neuroradiology: journal of peritherapeutic neuroradiology, surgical procedures and related neurosciences. 10.1177/15910199241298727 PMC11580117 39569567

[B53] Wang M. Mungur R. Lan P. Wang P. Wan S. (2018). MicroRNA-21 and microRNA-146a negatively regulate the secondary inflammatory response of microglia after intracerebral hemorrhage. Int. Journal Clinical Experimental Pathology 11 (7), 3348–3356. 31949711 PMC6962877

[B54] Weigel R. Schilling L. Krauss J. K. (2022). The pathophysiology of chronic subdural hematoma revisited: emphasis on aging processes as key factor. GeroScience 44 (3), 1353–1371. 10.1007/s11357-022-00570-y 35461468 PMC9213588

[B55] Yamashima T. (2000). The inner membrane of chronic subdural hematomas: pathology and pathophysiology. Neurosurg. Clinics N. Am. 11 (3), 413–424. 10918010

[B56] Yan S. Zhitao W. Li L. Dong W. Jianning Z. J. C. (2013). The level of circulating endothelial progenitor cells may be associated with the occurrence and recurrence of chronic subdural hematoma, 68(8) 10.6061/clinics/2013(08)04 PMC3752634 24037002

[B57] Yi X. Yue J. Yue S. Li J. Zhang X. Zhao G. (2025). The evolution and recurrence of chronic subdural hematoma was associated with different distribution of macrophage M1/M2 polarization. Inflammation 48, 4160–4171. 10.1007/s10753-025-02318-0 40397352 PMC12722378

[B58] Yokobori S. Sasaki K. Kanaya T. Igarashi Y. Nakae R. Onda H. (2019). Feasibility of Human neural stem cell transplantation for the treatment of acute subdural Hematoma in a rat model: a pilot Study. Front. Neurology 10, 82. 10.3389/fneur.2019.00082 30809187 PMC6379455

[B59] Yun H. J. Ding Y. (2020). How to remove those bloody collections: nonsurgical treatment options for chronic subdural hematoma. Brain Circ. 6 (4), 254–259. 10.4103/bc.bc_73_20 33506148 PMC7821810

[B60] Zhang Q. Chen R. Shi L. Zhao H. Yin F. Yu C. (2024). Single-cell sequencing analysis of chronic subdural hematoma cell subpopulations and their potential therapeutic mechanisms, Brain Res. Bull. 211 110936. 10.1016/j.brainresbull.2024.110936 38554980

[B61] Zhong D. Cheng H. Xian Z. Ren Y. Li H. Ou X. (2024). Advances in pathogenic mechanisms, diagnostic methods, surgical and non-surgical treatment, and potential recurrence factors of Chronic Subdural Hematoma: a review, Clin. Neurology Neurosurg. 242 108323. 10.1016/j.clineuro.2024.108323 38749358

